# Molecular investigation of bovine viral diarrhea virus infection in yaks (*Bos gruniens*) from Qinghai, China

**DOI:** 10.1186/1743-422X-11-29

**Published:** 2014-02-14

**Authors:** Xiaowei Gong, Lihong Liu, Fuying Zheng, Qiwei Chen, Zhaocai Li, Xiaoan Cao, Hong Yin, Jizhang Zhou, Xuepeng Cai

**Affiliations:** 1 State Key Laboratory of Veterinary Etiological Biology, Key Laboratory of Veterinary Public Health of Ministry of Agriculture, Lanzhou Veterinary Research Institute, Chinese Academy of Agricultural Sciences, No. 1 Xujiaping, Chengguan, Lanzhou 730046, People’s Republic of China; 2 Department of Virology, Immunobiology and Parasitology, National Veterinary Institute, 75189 Uppsala, Sweden

**Keywords:** Bovine viral diarrhea virus, Yak, 5′UTR, N^pro^, Phylogeny

## Abstract

**Background:**

Bovine viral diarrhea virus (BVDV) is a pestivirus which infects both domestic animals and wildlife species worldwide. In China, cattle are often infected with BVDV of different genotypes, but there is very limited knowledge regarding BVDV infection in Chinese yaks and the genetic diversity of the virus. The objectives of this study were to detect viral infection in yaks in Qinghai, China and to determine the genotypes of BVDV based on analysis of the 5′untranslated region (5′UTR) and N-terminal protease (N^pro^) region.

**Results:**

Between 2010 and 2012, 407 blood samples were collected from yaks with or without clinical signs in six counties of Qinghai Province. Ninety-eight samples (24%) were found to be positive by reverse transcription polymerase chain reaction (RT-PCR) targeting a conserved region of BVDV-1 and BVDV-2. The nucleotide sequences of the 5′UTR and complete N^pro^ region were determined for 16 positive samples. Phylogenetic reconstructions demonstrated that all 16 samples belong to subgenotypes BVDV-1b, BVDV-1d and BVDV-1q.

**Conclusions:**

This study provides, for the first time, molecular evidence for BVDV infection in yaks in Qinghai involving multiple subgenotypes of BVDV-1. This may have occurred under three possible scenarios: interspecies transmission, natural infection, and the use of vaccines contaminated with BVDV. The results have important implications for yak production and management in China, and specifically indicate that unscientific vaccination practices should be stopped and bio-security increased.

## Background

Bovine viral diarrhea virus (BVDV) is one of the most important viral pathogens of cattle worldwide. BVDV is classified into two species, namely BVDV-1 and BVDV-2, within the genus *Pestivirus*, which also includes border disease virus (BDV) and classical swine fever virus (CSFV) [1]. In addition, an atypical bovine pestivirus, or BVDV-3 [2], has recently been detected in cattle in South America, Asia and Europe and in contaminated Madin-Darby bovine kidney (MDBK) cells [3-7]. Based on comparisons of the sequences of 5′UTR, N^pro^ and E2 regions, BVDV-1 can be divided into 17 subgenotypes (1a-1q), and BVDV-2 into four subgenotypes (2a-2d) [8-13]. BVDV exists as two biotypes, designated cytopathic (CP) or noncytopathic (NCP) according to their ability to cause cytopathic effects in cell cultures [14]. The host range of BVDV is extensive, including cattle, sheep, goats, swine, yaks, deer and members of the Camelidae family [15,16]. Although cattle are generally believed to be the main source of BVDV infection, the prevalence of BVDV in the Chinese pig population has increased in recent years [17]. Bactrian camels have also been found to be infected with BVDV in western China [10]. Currently, nine subgenotypes are circulating among multiple hosts in China: BVDV-1a, BVDV-1b, BVDV-1c, BVDV-1d, BVDV-1 m, BVDV-1o, BVDV-1p, BVDV-1q and BVDV-2a [9,10,18-21].

The yak (Bos grunniens) is a unique bovine species that lives at high altitude (above 3,000 m) in China, Mongolia, Bhutan, Nepal, India, Russia, and other countries [22]. The yak population is estimated at around 14.2 million in total, of which 13.3 million yaks live in the Chinese territories and 5 million in Qinghai province alone. The ecosystem involving yaks in the Qinghai-Tibet Plateau is thought to be complex, because yaks share pastures and water with cattle, sheep, goats, camels, and even wild animals. This creates a great potential for interspecies transmission of pathogens such as BVDV. Indeed, a recent study has demonstrated infection of Bactrian camels with a BVDV strain that is closely related to the virus found in cattle [10]. Routine vaccination of yaks against bovine viral diarrhea using the CSFV C-strain live vaccine has been applied on some farms in this region since the early 1980s [23,24]. Such a practice might have introduced more problems for the prevention and control of diseases in the yak.

Since the first serological detection of BVDV infection in yaks in Qinghai, China [25], numerous investigations have documented a seroprevalence of BVDV antibodies ranging from 0.56% to 72.14% [26-29]. While a molecular epidemiological survey of BVDV in yaks has been conducted in the Himalayan region [30], it is unclear if a similar situation of BVDV infections in the Chinese yaks. The objectives of this study were to gain knowledge and to better understand the complex nature of BVDV infection in yaks in western China.

## Results and discussion

A 230-bp fragment of the 5′UTR and the entire 504-bp N^pro^ coding region were amplified by RT-PCR from RNA extracted from blood samples. A total of 98 (24%) out of 407 blood samples were BVDV positive. The percentages of BVDV-positive samples were 20.5% (16/78) in Yushu, 18.8% (13/69) in Zhiduo, 35.5% (16/45) in Zeku, 35.2% (30/85) in Dari, 16.9% (10/59) in Xinghai, and 18.3% (13/71) in Dulan.

In western China, BVD has been reported previously in cattle and Bactrian camels with a prevalence of 43% and 46.4%, respectively [10,18]. Given that yaks, cattle and even camels live close together, they may have direct and indirect contact each other, allowing easy transmission of BVDV among them. However, serological tests may not reflect BVDV infection in yaks reliably because of unscientific use of CSFV C-strain live vaccine in yaks in these regions. Therefore, molecular investigation within the BVDV-infected herds, nucleotide sequencing and phylogenetic analysis will contribute to a better understanding of the evolutionary pathways of BVDV infection.

**Figure 1:**
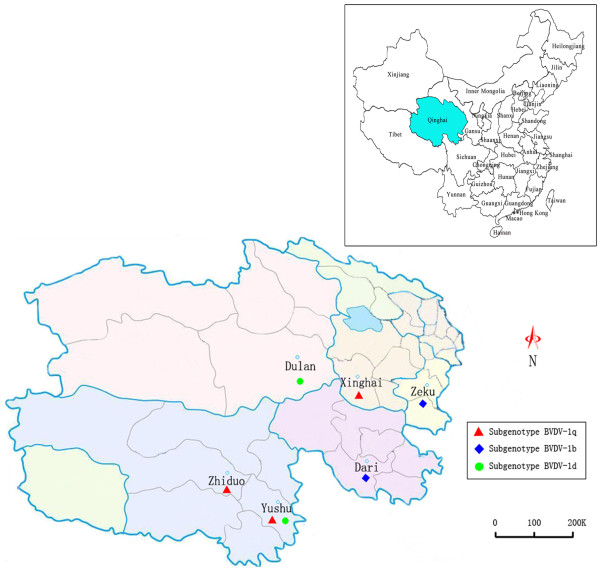
Geographical distribution of BVDV subtypes circulating in six counties of Qinghai.

**Figure 2:**
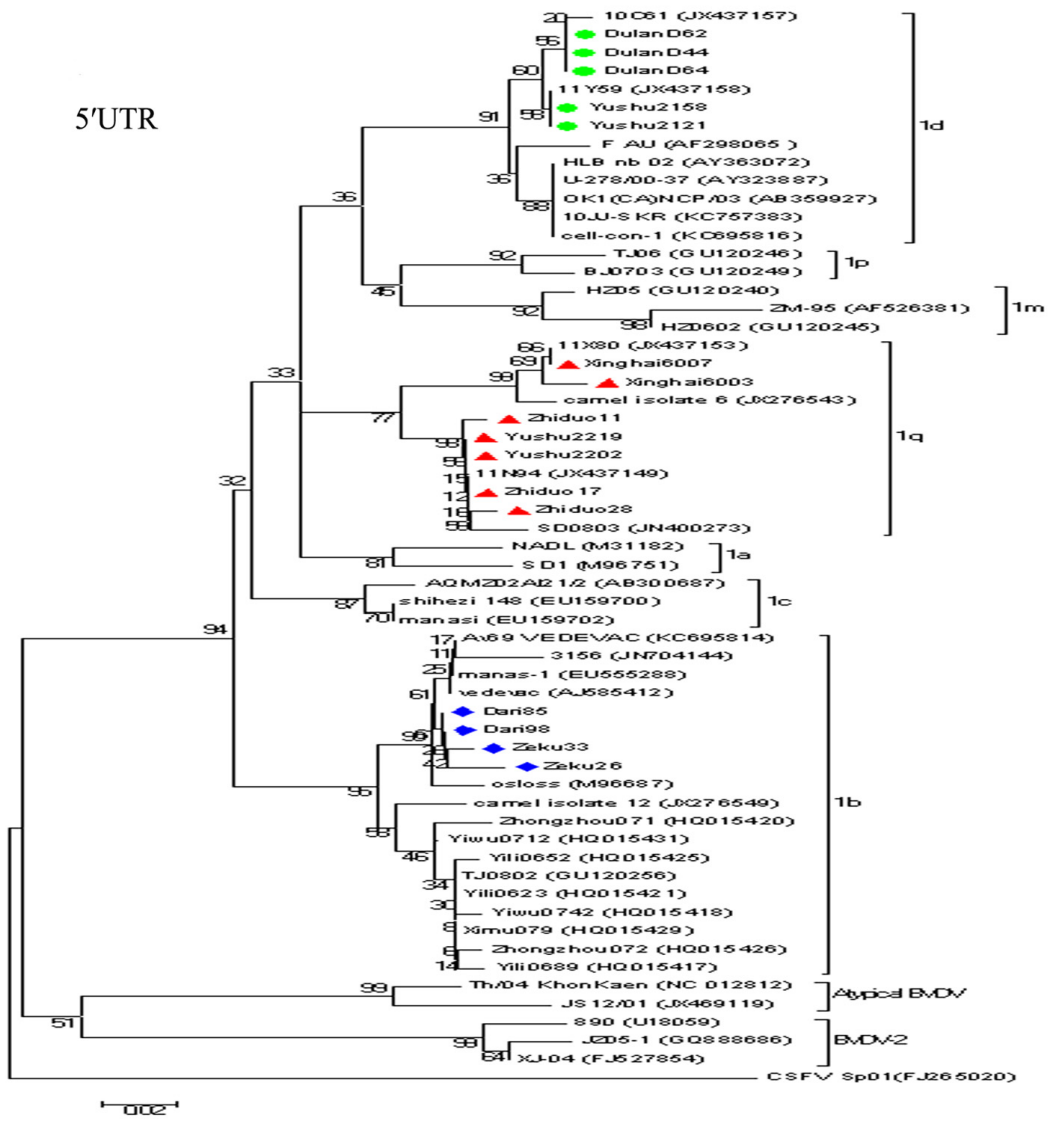
**Unrooted phylogeneitc tree based on the 5′UTR sequences.** Phylogenetic tree of the 5′UTR from 16 BVDV positive samples was constructed by the neighbor-joining (NJ) method (Kimura two-parameter method) with the sequences published in GenBank. The nucleotide length of the 5′UTR used for the analysis was 230 bp. The numbers at the phylogenetic branches indicate the bootstrap values (1000 replicates) in percentage supporting each group. The bar represents a genetic distance.

**Figure 3:**
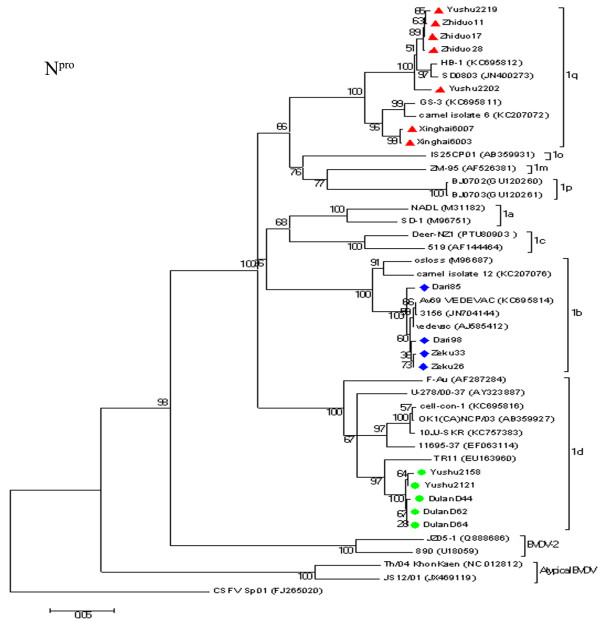
**Unrooted phylogeneitc tree based on the N**^**pro **^**sequences.** Phylogenetic tree of the N^pro^ region from 16 BVDV positive samples were constructed by the neighbor-joining (NJ) method (Kimura two-parameter method) with the sequences published in GenBank. The nucleotide length of the N^pro^ used for the analysis was 504 bp. The numbers at the phylogenetic branches indicate the bootstrap values (1000 replicates) in percentage supporting each group. The bar represents a genetic distance.

Sixteen positive samples were selected from the six counties for further PCR amplification and sequencing of both the 5′UTR and the N^pro^ region. The nucleotide sequences of the 5′UTR and N^pro^ region have been deposited in GenBank under the following accession numbers: KC414598 - KC414613 and KC414582 - KC414597. Blast analysis of the 5′UTR and N^pro^ sequences showed that all 16 samples belonged to BVDV-1. The nucleotide sequence similarities of the N^pro^ between these positive samples and the reference strains were 78.0-99.2% for BVDV-1 (NADL, Osloss, VEDEVAC), 68.8-71% for BVDV2 (890, JZ05-1), and 63.7-67.3% for atypical bovine pestivirus (TH/04_KhonKaen, JS12/01 strain), respectively. Phylogenetic analysis showed that the yaks were infected with viruses of three subgenotypes: BVDV-1b (four samples), BVDV-1d (five samples) and “BVDV-1q” (seven samples), as shown in Figures 1, 2 and 3. Two subgenotypes, BVDV-1d and -1q, were identified in the same county, indicating multiple origins of the viruses. Further sequencing of more viruses might identify this pattern in other counties. Regardless of the genetic region, both phylogenetic trees produced consistent results, although the N^pro^ phylogenetic tree showed highest bootstrap values (over 95%). BVDV-2 was not detected in yaks in this study.

Seven yaks from three counties were infected with BVDV-1q. Unexpectedly, the viral sequences from five yaks in Yushu and Zhiduo counties shared a close relationship with BVDV strain SD0803 of porcine origin in Shandong province and 11N94 of cattle origin in Ningxia Hui Autonomous Region (Figure 2). SD0803 was isolated from a diseased pig in China [31], and five other closely related viruses were found in three provinces of China [19]. It has been suggested that vaccination of pigs with a BVDV-contaminated C-strain live vaccine might be an important route of infection in pigs, as BVDV contamination in the C-strain vaccine has been reported in China [32]. There are no accurate data on the cattle from which the BVDV 11N94 was detected, and therefore it cannot be established definitively whether the 11N94 infection occurred naturally or through contaminated biological products. Clustering of the two viruses with camel-6 of camel origin and 11X80 of bovine origin indicated interspecies transmission; however, the direction of transmission is unknown.

BVDV-1b is considered to be a predominant BVDV-1 subgenotype circulating in Chinese cattle [9,18]. Surprisingly, four yaks from two counties were infected with BVDV-1b strains that were closely related to sequences from a BVDV vaccine Vedevac, with strong support in both the 5′UTR and N^pro^ phylogenies. Vedevac was found as the main virus strain in batches of BVDV vaccine Oregon C24V in Hungary, and it was likely to have been picked up accidently during passages of the original Oregon C24V for vaccine development between the 1960s and 1990s [33]. In China, the Oregon C24V vaccine has been distributed to different laboratories as a reference strain for BVDV, but it has never been used for vaccination against BVDV in yaks in this region. The 16 samples in this study had not been passaged in cell culture. Therefore cell culture contamination is unlikely to be the origin of the viruses found. The phyologenetic positioning of Vedevac among the four yak BVDV strains and additional cattle BVDV sequences in the 5′UTR tree suggests that BVDV strains circulating in yaks and other animal species in Western Asia/Eastern Europe may represent gene pools where the Vedevac derived from.

Yaks in Yushu and Dulan counties were infected with BVDV-1d, which further demonstrates the complex nature of BVDV infection in the Chinese yaks. BVDV-1d infection in cattle has been reported previously in Ningxia Hui Autonomous Region [20], and also in cell cultures contaminated with a virus named “cell-con-1” (GenBank no. KC695816). In addition, BVDV-1d is common in central European countries including Poland, Austria, Germany, Italy, the Czech Republic, Slovenia and Turkey, and East Asian countries such as Japan [13,34-40].

## Conclusion

This report has demonstrated that yaks in the western China are infected with multiple subgenotypes of BVDV-1 under several possible scenarios such as interspecies transmission, vaccination with contaminated CSFV C-strain vaccine and natural infection. The BVDV circulating in yaks and other animal species in Western Asia/Eastern Europe may be the genetic source for viruses found in the BVDV vaccine strain Oregon C24V. Further studies are required to elucidate the exact origins of BVDV transmission in yaks. Finally, the results suggested the importance of urgently correcting unscientific vaccination practices in yak production in China.

## Materials and methods

### Ethics statement

Before blood samples collection, we contacted local veterinary practitioners and the owners of the animals and obtained their permission. All the experimental protocols were approved by the Animal Ethics Committee of Lanzhou Veterinary Research Institute, Chinese Academy of Agricultural Sciences, Lanzhou, China.

### Samples

Blood samples from 407 yaks in six counties of Qinghai Province (31°45′-36°10′N, 89°27′-97°39′E), namely Yushu, Zhiduo, Dari, Zeku, Xinghai and Dulan, were used for this study (Figure 1). The samples were collected by local veterinary practitioners and transported to our veterinary diagnostic laboratory at low temperatures during August 2010 to January 2012. The yak population of these regions represents 25% of all yaks in Qinghai. According to local veterinarians, routine vaccination with CSFV live vaccine was performed in about 15%-20% of the yaks in the sampled areas, and there were no precise data available on the use of other live vaccines. A total of 13 tested animals from Zeku county exhibited clinical signs of diarrhea, while the rest were clinically healthy at the time of sampling.

### RNA extraction and cDNA synthesis

RNA was extracted from whole blood using a QIAamp viral RNA mini kit (Qiagen, USA). Virus cDNA was constructed by reverse transcription performed using an M-MLV first strand kit (Invitrogen, Shanghai, China) with random primer in accordance with the manufacturer’s instructions. The cDNA samples were cooled to 4°C, and then either used directly for PCR or stored at -20°C.

### RT-PCR and nucleotide sequencing

In order to determine the nucleotide sequences of the BVDV samples, two sets of common primers for BVDV-1 and BVDV-2 (F1 5′-GCCATGCCCTTAGTAGGACT-3′, and R1 5′-CACCCTATCAGGCTGTRTYC-3′, F2 5′-CTCTGCTGTACATGGCACAT-3′ and R2 5′-GAGCAGCTKGTGACCCATAR-3′) were designed on the basis of the conserved regions of reference strains. The primer pair F1/R1 resulted in a 230-bp fragment of the 5′UTR after amplification whereas the primer pair F2/R2 amplified the product containing entire N^pro^ region. The PCR amplification was conducted in a total volume of 25 μl containing 12.5 μl Premix Ex Taq (TaKaRa, Dalian, China), 2 μl cDNA, 9.5 μl sterilized H_2_O, and 0.5 μM each of the primers. The PCR cycling involved 94°C for 5 min, followed by 30 cycles at 94°C for 45 s, 61°C for 45 s and 72°C for 1 min, with a final elongation step at 72°C for 10 min. The amplified products were analyzed by 1.5% agarose gel electrophoresis. Sixteen samples which represented six counties in Qinghai were chosen from among the 98 positive samples for sequencing. The PCR products were purified with the TaKaRa Agarose Gel DNA Purification Kit Ver.2.0 and were cloned into the pMD-18 T vector (TaKaRa, Dalian, China). The recombinant plasmids were sequenced with the primers M13F and M13R at the Nanjing GenScript Biotech Co., Ltd., Nanjing, China.

### Phylogenetic analysis

Sequence data were assembled and analyzed using the DNAStar version 7.0 package (DNAStar Inc., USA). Multiple sequences were aligned using the CLUSTALW program with the corresponding regions of BVDV-1, BVDV-2, atypical BVDV and CSFV reference sequences retrieved from GenBank. Phylogenetic reconstructions for genotyping were compiled using the 230-bp fragment of the 5′UTR and the entire 504-bp N^pro^ coding region, and phylogenetic trees were constructed under Kimura two-paramer model using MEGA version 4.1. The reliability of the neighbor-joining tree was estimated by boostrap analysis with 1,000 replicates.

## Competing interests

The authors declare that they have no competing interests.

## Authors’ contributions

XG designed the experiments for sequence and did the phylogenetic analysis, and drafted the manuscript. XC and HY contributed to the conception and design of the study. JZ and QC helped in the contacting veterinary practitioners of Qinghai and did the sample collection. FZ, XC and ZL performed the RT-PCR tests. LL contributed to data analysis and revision of the manuscript. All authors read and approved the final manuscript.
